# Draft Genome Sequence of *Bacillus amyloliquefaciens* JJC33M, Isolated from Sugarcane Soils in the Papaloapan Region, Mexico

**DOI:** 10.1128/genomeA.01519-14

**Published:** 2015-02-12

**Authors:** J. J. Montor-Antonio, B. Sachman-Ruiz, L. Lozano, S. del Moral

**Affiliations:** a Instituto de Biotecnología, Universidad del Papaloapan, Tuxtepec, Oaxaca, Mexico; b Centro Nacional de Investigación Disciplinaria en Parasitología Veterinaria del Instituto Nacional de Investigaciones Forestales Agrícolas y Pecuarias, Jiutepec, Morelos, Mexico; c Centro de Ciencias Genómicas, Universidad Nacional Autónoma de México, Cuernavaca, Morelos, Mexico

## Abstract

Bacillus amyloliquefaciens strain JJC33M is a bacterium that produces α-amylase (EC 3.2.1.1) and was isolated from sugarcane soil. Its estimated genome size is 3.96 Mb, and it harbors 4,048 coding genes (CDSs).

## GENOME ANNOUNCEMENT

A draft genome of *Bacillus amyloliquefaciens* strain JJC33M was obtained by direct sequencing of genomic DNA using Illumina sequencing technology. We isolated *B. amyloliquefaciens* JJC33M from sugarcane soil in the Papaloapan region in Mexico. *B. amyloliquefaciens* JJC33M produces an α-amylase (EC 3.2.1.1) not dependent on calcium, which may give advantages in the production of syrups; it also has the capability of being stable at 40°C, which means it could be used in the baking industry (1). Recently, the genomes of *B. amyloliquefaciens* DSM7, UCMB5036, and EBL11, which are strains used in industry, have been sequenced (2, 3).

JJC33M was grown on nutrient agar with 1% (P/V) starch medium in a petri dish. The genomic DNA was prepared by a MoBio microbial DNA isolation sample prep kit and sequenced by an Illumina HiSeq 2000 sequencer at Macrogen, Seoul, South Korea. A total of 10.19 million paired-end reads with length of 101 bp were obtained from the sequencing. The raw reads were initially assembled using SPAdes version 3.1.1. The gaps between the contigs and ambiguous patches within the contigs were manually filled by aligning individual reads to the contig ends using in-house programs.

The genome sequence annotation was performed with RAST (4). The draft genome of *B. amyloliquefaciens* JJC33M contains 40 contigs, with a total length of 3,961,663 bp. The genome contains 4,048 protein-coding genes (CDSs), 53 rRNAs, and 89 tRNAs, with a G+C content of 45.7%. Genome analysis revealed that the genome of *B. amyloliquefaciens* JJC33M has a high similarity to that of strain *B. amyloliquefaciens* IT-45 (identity, 94.57%).

### Nucleotide sequence accession numbers.

This whole-genome shotgun project has been deposited at DDBJ/EMBL/GenBank under the accession no. JTJG00000000. The version described in this paper is version JTJG01000000.
